# A Validation Approach for Quasistatic Numerical/Experimental Indentation Analysis in Soft Materials Using 3D Digital Image Correlation

**DOI:** 10.3390/ma10070722

**Published:** 2017-06-28

**Authors:** Luis Felipe-Sesé, Elías López-Alba, Benedikt Hannemann, Sebastian Schmeer, Francisco A. Diaz

**Affiliations:** 1 Departamento de Ingeniería Mecánica y Minera, Campus las Lagunillas, Universidad de Jaén, 23071 Jaén, Spain; elalba@ujaen.es (E.L.-A.); fdiaz@ujaen.es (F.A.D.); 2 Institute for Composite Materials (IVW), Kaiserslautern University of Technology, 67663 Kaiserslautern, Germany; benedikt.hannemann@ivw.uni-kl.de (B.H.); sebastian.schmeer@ivw.uni-kl.de (S.S.)

**Keywords:** Digital Image Correlation, instrumented indentation, numerical validation, Image Decomposition, strain analysis

## Abstract

A quasistatic indentation numerical analysis in a round section specimen made of soft material has been performed and validated with a full field experimental technique, i.e., Digital Image Correlation 3D. The contact experiment specifically consisted of loading a 25 mm diameter rubber cylinder of up to a 5 mm indentation and then unloading. Experimental strains fields measured at the surface of the specimen during the experiment were compared with those obtained by performing two numerical analyses employing two different hyperplastic material models. The comparison was performed using an Image Decomposition new methodology that makes a direct comparison of full-field data independently of their scale or orientation possible. Numerical results show a good level of agreement with those measured during the experiments. However, since image decomposition allows for the differences to be quantified, it was observed that one of the adopted material models reproduces lower differences compared to experimental results.

## 1. Introduction

Today, the investigation of engineering problems involving the use of large deformation materials such as silicon, rubber, or biological materials has focused the attention of many researchers [1]. One typical field of research for such materials is the analysis of their contact behaviour during indentation experiments. These materials exhibit large displacements under contact loading due to their physical behaviour. One major difficulty in the analysis of contact problems in hyperplastic materials is that their mechanical behaviour cannot always be described by theoretical models [2]. Some analytical [3,4,5,6,7,8,9] and numerical [10,11,12] studies can be found in the literature that have contributed to a better knowledge of such a problem, but they often consider different assumption for their analysis, such as assuming small strains below the elastic limit [6,8,11], considering half-plane elastic for both the indenter and the soft body material [7,9,11,12], assuming that the contact area is much smaller than the radius of the indenter and the elements are frictionless [7,10,11], or that the external angle of the indenter is very small [8]. Nevertheless, some of these assumptions are not always well supported by lab experiments. Although experimental techniques constitutes an important alternative to validate analytical models and numerical studies, no substantial work can be found in the literature [2,13,14,15,16] for the analysis of large deformation materials.

In this paper, one of the best established optical techniques for full field displacement measurement is applied to the analysis of large strains due to indentation in a hyperplastic material. The adopted technique is 3D digital image correlation (3D-DIC) [17], which employs at least two cameras for a stereoscopic visualization of the surface of the specimen with a random distribution of light intensity (speckle). The element surface can be unambiguously distinguished by its random neighbourhood pattern. In this way, a group of pixels corresponding to a specific location at the element surface is identified on a sequence of images captured during deformation with the aid of an image correlation algorithm [17]. Displacement fields of the studied surface are obtained, and from them, strain maps can be also calculated. 

3D-DIC is employed for the analysis of contact experiments using a wedge-shape indenter on a soft material cylinder. The experiment was performed to obtain displacements and strains fields. Two analytical studies employing Finite Element Method representing the experimentation were also performed. Those analyses employed two different mathematical formulation to determine the material behaviour.

During the experiments, it was not possible to guarantee avoiding solid rigid displacement, which could affect to the displacement measurements with 3D-DIC. For that purpose, it is believed that strain maps more adequately represent the mechanical behaviour of the material during the test rather than displacement maps. In order to evaluate the accuracy of the numerical simulation performed using FEM models, strain maps from the experiments and FEM were compared. The compassion of strain maps obtained by employing different methodologies is a challenging task. In this paper, a novel methodology based on the decomposition of the strain maps from both experiment and simulation was evaluated to perform quantitative comparisons of the indentation process at different displacement steps. The adopted comparison methodology is based on an Image Decomposition algorithm [18]. It consists of the decomposition of each data field into a feature vector that is independent from scale or orientation of the original data map. It is necessary to decompose each of the displacement maps captured along the test in order to study an event through time. Nevertheless, this paper presents a novel procedure to encode the full experimentation in a single strain map (based on Tchebichef shape descriptors [19]). This procedure decreases the dimensionality of the comparison process compared to some previous work [2,20]. 

## 2. Methodology

### 2.1. Digital Image Correlation

**Figure 1:**
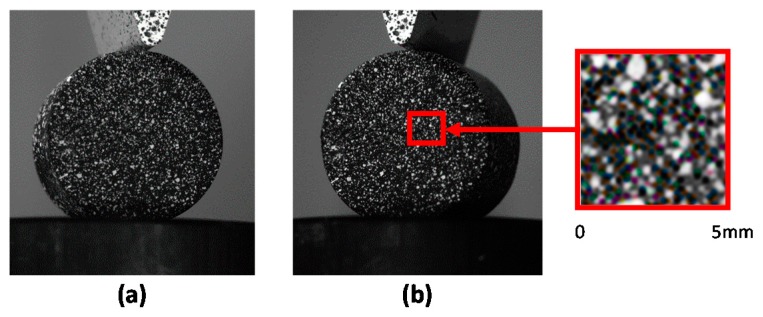
Image captured by left camera (**a**) and right camera (**b**) of a stereoscopic DIC system.

The full field technique employed for the measurement of strains at the surface of the specimen was Digital Image Correlation 3D (3D-DIC). This technique requires a stereoscopic digital camera system acquiring images from two different points of view of the area of interest in order to measure the 3D shape and displacements occurring at that surface as observed in Figure 1. Additionally, the surface should present a randomly distributed speckle pattern to ease the tracking of the points that compose that region of interest. This is usually composed of white speckles over a black background (as observed in the detail of Figure 1). DIC divides each of the images into small subsets [21], as observed in Figure 1. Every subset has a unique intensity pattern within the subset due to the randomness of the speckle pattern. 

The most similar intensity pattern of this subset in the first speckle image is then searched for in a second speckle image in an area around the same pixel position. Once the intensity pattern of the subsets closely coincides, the displaced pixel is found. Since two cameras are observing the same area of interest, a calibration procedure is required to accurately determine the three-dimensional position of the subsets. This calibration procedure consists of the calculation of intrinsic (focal length, image size, aberration of lenses) and extrinsic (related to the position of the cameras and the specimen) parameters of the optical set-up [22,23]. 

### 2.2. Experimental Set-Up for Contact Experiments 

The experimental work presented consisted of the indentation of a cylinder made of a black rubber material employed in automotive applications. The material cannot be disclosed for confidentially reasons, although this does not affect the aim of the paper, which is the validation of the procedure. The shape of the specimen was 15 mm in height and 25 mm in diameter, and the contact area was lubricated with oil. 

**Figure 2:**
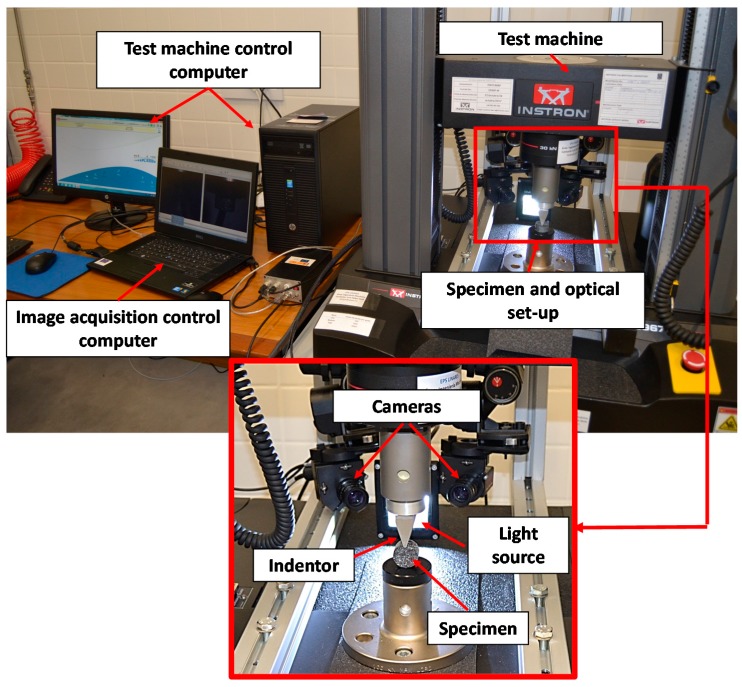
Illustration of the experimental set-up with detail of the optical arrangement.

**Figure 3:**
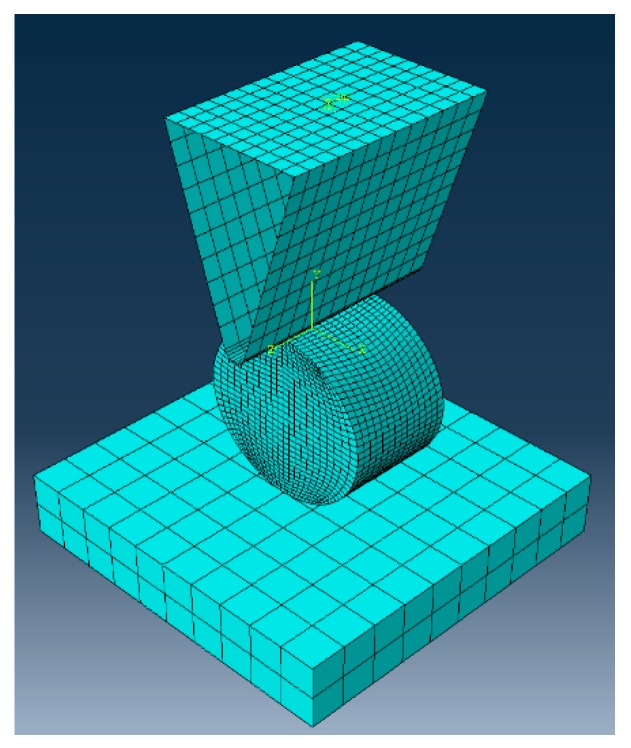
Detail of the finite element mesh employed for numerical modeling.

The experimental set up is shown in Figure 2. For indentation, a 2024 aluminum wedge was employed as illustrated in Figure 3. To control the displacement of the indenter, an Instron testing machine (model 5967 with 30 kN load capacity) was employed. The indentation was achieved by performing a displacement of the indenter from 0 mm to −5 mm and then unloading the indenter back to 0mm. The speed of the displacement was set to 10 mm/min.

The full-field response of the specimen during the indentation was captured by a stereoscopic system composed of two CCD monocromatic cameras (brand Allied model Stingray) with 2452 × 2056 pixels resolution and a 35 mm f 1.6 focal length lenses (brand Goyo Optical Inc., Saitama, Japan). Since the rubber material was completely black, the speckle pattern was obtained by spraying white paint over the surface of the specimen. During the tests, two images were synchronously captured by the stereoscopic camera system at different indentation steps, from 0 mm to −5 mm and back to 0 every 0.5 mm increment (a total of 21 steps and 42 images). The trigger signal was commanded by the testing machine and was registered by VicSnap commercial software from Correlated Solutions.

The calibration was performed employing a 12 × 9 targets with 5 mm spacing calibration target supplied with the commercial software VIC 3D (Correlated Solutions).

Since DIC only measure 3D displacements maps, strains maps require a postprocesing analysis. In this case, strain maps were calculated employing a Lagrange Tensor and using a subset size of 15 pixels to obtain horizontal (ε_xx_) and vertical (ε_yy_) strains.

### 2.3. Numerical Modelling

An explicit numerical analysis was conducted using Abaqus 6.14 (commercially available software) for a 25 mm diameter rubber cylinder 15 mm high under lateral indentation at 10 mm/min using a 20 mm thick aluminum wedge with a tip radius of 1.68 mm. An included angle of 73.45° was also employed, as shown in Figure 3. 

The indenter was modelled as a non-deformable body using 520 bidimensional rigid solid elements (type R3D4). The rubber cylinder was modelled using 9800 solid reduced integration elements (type C3D8R). The size of these elements varied according to their position in the cylinder from 0.5 mm to 1 mm. The minimum element size controlled the minimum time-step in the explicit solution. The values were chosen to give an appropriate temporal and spatial resolution. Additionally, friction coefficient was estimated to be 0.5, according to previous studies [24,25].

The rubber is known to exhibit a non-linear elastic behavior that can be modelled using different models [26]. In this paper, two different materials models are evaluated. The first material model (FEM Model A) is Neo-Hookean [27]: the expression of the material’s strain energy function is described by Equation (1). The second (FEM Model B) is Van der Waals [28], which follows Equation (2).

(1) $$W=C_{1}\left(I_{1}-3\right),$$

(2) $$W=\mu c_{e}\ln\left(1-\sqrt{\frac{I_{1}-3}{c_{e}}}\right)-\mu c_{e}\sqrt{\frac{I_{1}-3}{c_{e}}},$$

where *W* is the strain-energy density, µ is the shear modulus, *C*_1_ is a material constant, *I*_1_ is the first invariant of the right Cauchy-Green deformation tensor, and *c_e_* is the chain extensibility dependent of the material.

**Figure 4:**
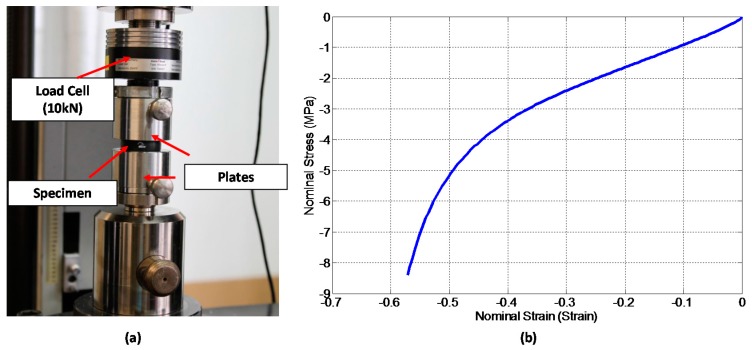
Illustration of compression test. (**a**) Image of the specimen during compression test; (**b**) Stress-Strain response of the rubber.

The material model parameters were obtained by providing empirical data from the compression tests that were used by the Abaqus subroutine to evaluate the response of the material. The uniaxial compression tests were performed on a similar cylinder on which the experimental indentation where performed but with metallic cups on the extremes of the cylinder to homogenize the compression force. The material test specimen was loaded between platens at 10 mm/min up to a compression of 12 mm using a Zwick 1474 machine and performing four cycles of pre-conditioning up to 13 mm compression. An illustration of the compression test and the results from these tests are shown in Figure 4. 

The applied load was obtained from the load cell of the test machine and used to compute engineering stress by dividing the applied load by the original cross-section area of the specimen (1.963 × 10^−3^), while the displacement of the cross-head of the test machine was used to evaluate engineering strain by dividing the measured displacement by the original height of the specimen. Data in Figure 4b were employed to evaluate the material parameters to satisfy Equations (1) and (2) using the EDIT MATERIAL tool from Abaqus/CAE 6.14-2. The movement of the wedge into the cylinder and the release of the wedge after indentation were both modelled using wedge speeds obtained from the experiments measured by applying speckle pattern on the indenter as illustrated in Figure 1 and Figure 2.

### 2.4. Validation Procedure

The amount of experimental and numerical results for the 21 indentation steps—a total of 126 strain maps considering 21 studied steps employing three different methods (two numerical and one experimental) and two different studied strain maps (ε_xx_ and ε_yy_)—was difficult to manage. Additionally, experimental and numerical results were not directly comparable due to the different angle of view of the cameras and differences in the scale of the data exportation files. However, a comparison between both data sets was required, so an image decomposition method was employed [18]. This method was employed for the validation of the analytical and theoretical models, and its fundamentals consisted of converting the information from strain maps into a feature vector that can be directly compared for each indentation step. The more similar the vectors, the more similar are the results. Using this approach, it is possible to eliminate the influence of the images’ size and camera view angles, making it possible to perform a direct comparison of the results. 

The adopted image decomposition method was based on Tchebichef polynomials *T*(*i*,*j*) [19] to decompose displacements images **I**(*i*,*j*) into shape descriptors that have the same units of the decomposed strain map (in this case was units of strain mm/mm).

(3) $$I\left(i,j\right)=\overset{N}{\underset{k=0}{\sum }}s_{k}T_{k}\left(i,j\right),$$

where the coefficients *s_k_* are called as feature or shape vector for the displacements map **I**(*i*,*j*) and they are determined by:

(4) $$s_{k}=\overset{N}{\underset{k=0}{\sum }}I\left(i,j\right)T_{k}\left(i,j\right),$$

**Figure 5:**
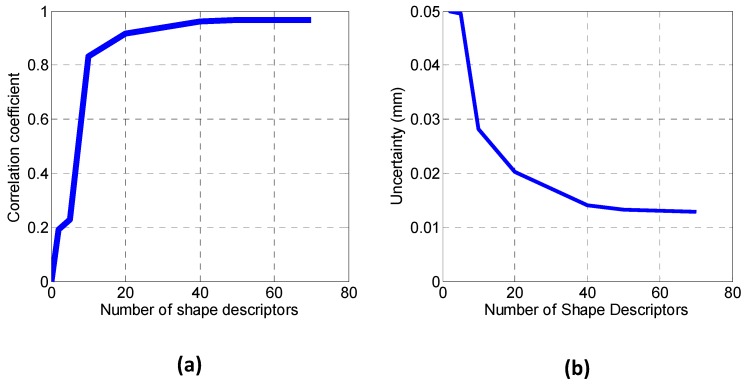
(**a**) Evolution of the correlation coefficient between original and reconstructed strain map and (**b**) Evolution of the Uncertainty of the reconstruction against the number of shape descriptors employed in the image decomposition.

Polynomials are dimensionless, and *N* is the number of moments or shape descriptor. The applicability of this image decomposition method is reduced to rectangular data fields. The required number of shape descriptors (SD) is given by a correlation coefficient between the original and the reconstructed image using N shape descriptors. Thus, a study of the adequate number of descriptors is required. As shown at Figure 5a, as the number of descriptors increases in the horizontal axis, the correlation coefficient (on the right ordinate axis) increases. Figure 5b indicates that as the number of moments increases, the calculated uncertainty (defined as the squared root of the squared difference between the original and the reconstructed images) decreases on the ordinate axis. Finally, for vectors comparison, 50 was considered as an appropriate number of shape descriptors, which is the quantity that raises the asymptotic maximum of the correlation coefficients in Figure 5.

**Figure 6:**
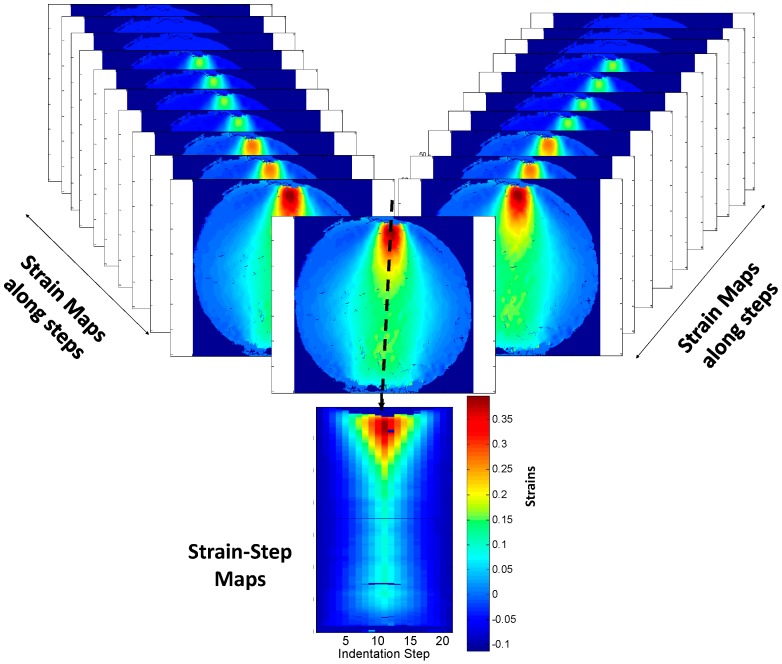
Illustration of the sampling of the strain profiles.

Nonetheless, it is necessary to compare feature vectors from ε_xx_ and ε_yy_ strains maps from 21 steps, and from experiments and a similar number of them for numerical results that results in 126 feature vectors. In this paper, an alternative comparison process was performed to minimize the quantity of data to compare and to make it possible to employ Tchebichef descriptors with circular strain maps. Hence, the evolution of a vertical and horizontal profile of horizontal (ε_xx_) and vertical (ε_yy_) strains maps along time in order was studied in order to encode the evolution in time in a single data field instead of 21 images. Data fields (strains-step maps) composed of 21 columns that correspond to vertical profiles (in the indentation direction) of the strain map along the mid-plane of the specimen at each of the 21 indentation steps were generated, as illustrated in Figure 6. This methodology was applied for ε_xx_ and ε_yy_ strains. Thus, one single image encodes ε_xx_ information and another image encodes ε_yy_ information over the whole test. Additionally, in order to compare the behavior in the normal direction to the load, the same procedures were followed, but in this case, the columns of the image consisted on horizontal profiles of the strain maps along the horizontal mid-plane of the specimen. This procedure makes it possible to compare only 12 strain-time or strain-step maps, six of them representing the vertical profiles of ε_xx_ and ε_yy_ for each of the three set of results obtained, and another six representing the horizontal profiles of those results. This leads to 12 feature vectors instead of 126.

## 3. Results

The experimental and numerical results of the indentation performed on the rubber cylinder are presented here together with the results of the comparative employing image decomposition. As mentioned above, the large amount of displacement and strain maps makes it necessary to reduce the presented data in order to accelerate the their direct comparison.

**Figure 7:**
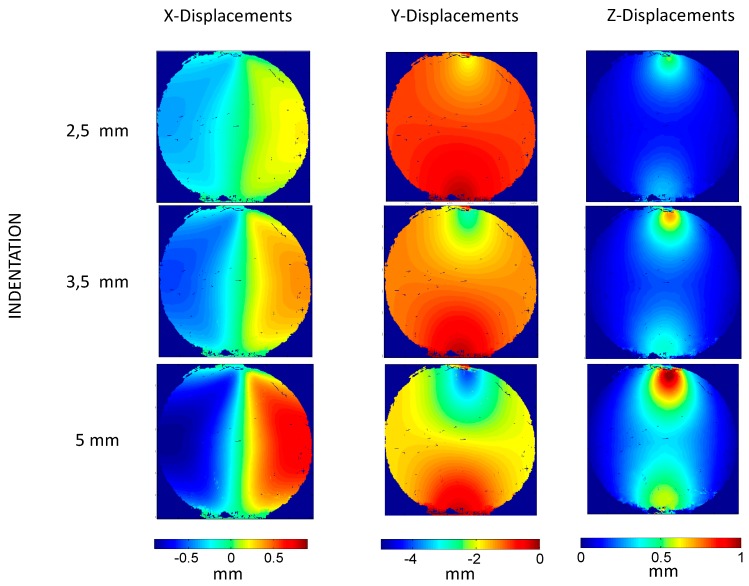
Displacement maps obtained experimentally at 2.5 mm (first row), 3.5 mm (second row) and 5 mm (third row) indentation obtained in Xcdirection (first column), Y direction (second column), and Z direction (third column).

For illustration purposes, Figure 7 shows the displacement maps obtained at three different indentation steps (i.e., 2.5, 3.5, and 5 mm) in the three spatial directions. As observed, there are some areas where the results were not successfully monitored due to the large level of deformation achieved. That is especially notorious in the upper area, where the contact with the indenter occurred, and in the lower area, where the specimen was supported.

In addition, some tilting on the displacement maps was also observed. This orientation was due to the angles required in the stereoscopic camera system to perform 3D-DIC. Nonetheless, the orientation of the axis coordinate system was selected to have a Y-axis in the direction of the indention and a perpendicular X-direction in order to have the same coordinates systems for both the experimental and FEM results.

**Figure 8:**
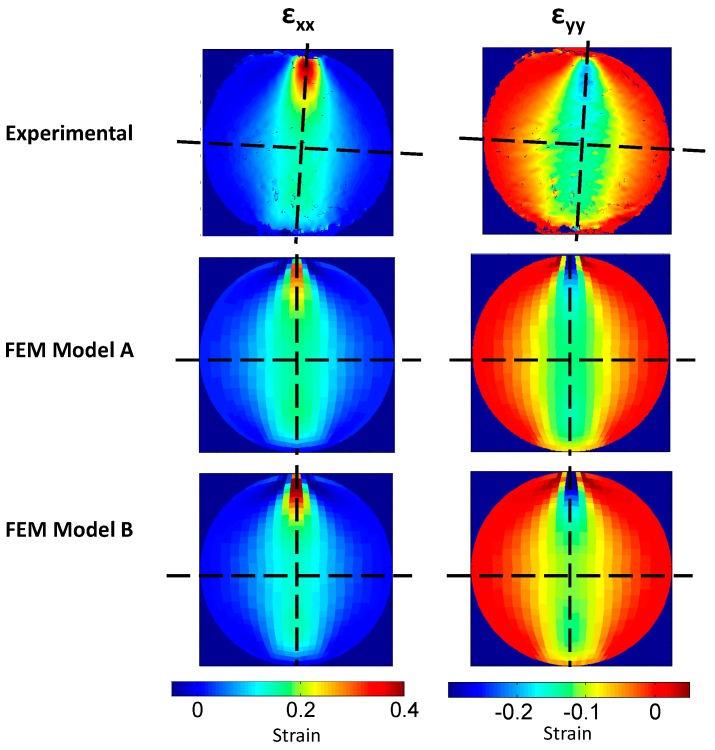
Strain maps in X direction (considered normal to indentation) and Y direction (considered in the direction of the indentation) Obtained experimentally (upper row), and numerically with FEM A and B (central and lower rows).

As previously mentioned, the focus was placed on the strain fields instead of the displacement fields to avoid possible solid rigid displacements and to analyze the material mechanical behavior. Hence, Figure 8 presents the experimental and numerical strain maps in X and Y directions (ε_xx_ and ε_yy_ respectively) obtained at maximum indentation of 5 mm on the rubber specimen.

As observed, strain maps obtained with FEM are very close to those obtained during the experiments. However, it is observed that no direct quantitative full-field comparison is possible due to the differences in size of the strain map and the orientation of the experimental strain maps. 

**Figure 9:**
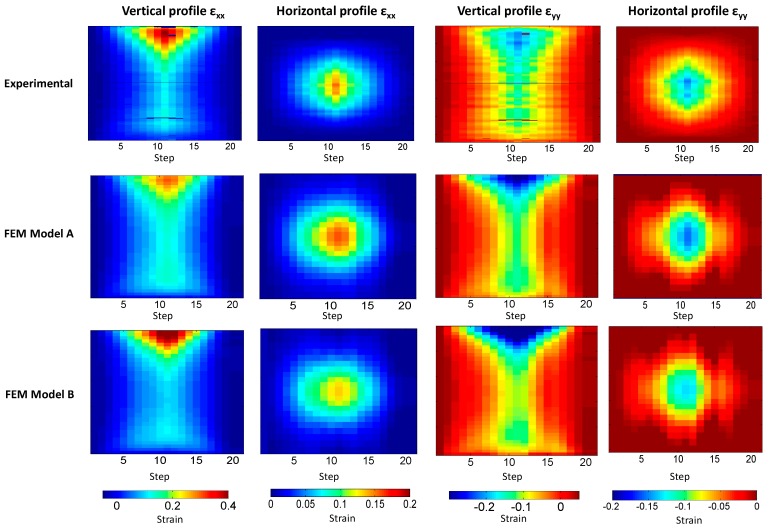
Strain-step maps obtained for ε_xx_ along vertical profile (first column); ε_xx_ along horizontal profile (second column); ε_yy_ along vertical profile (third column) and ε_yy_ along horizontal profile (fourth column) from experimental (first row), FEM A (second row) and FEM B (third row) results.

To overcome these drawbacks, the vertical and horizontal profiles are presented in Figure 8 as black dashed lines. The data of 126 strain maps considered in this study are simplified into 12 data fields encoding the strain data (strains-step maps) along the 21 steps, as presented in Figure 9.

A good correlation between experimental and numerical results is observed in Figure 9. However, some slight differences are present, and some quantification of them is required to define which numerical model represents the experimentation more adequately. 

Feature vectors make it possible to quantify differences by calculating the Euclidean distance between them. The Euclidean distance is simply the straight-line distance between the locations represented by the vectors in a multi-dimensional space, so that two coincident vectors have a Euclidean distance of zero. Table 1 shows the differences obtained between the strain-step maps presented in Figure 9.

The differences are in a lower order of magnitude of one tenth of the maximum strain value. Since feature vectors have the same length, a correlation coefficient between experimental and both numerical results was also calculated. This concordance correlation coefficient provides an indication of the extent to which the components of the feature vector fall on a straight line of gradient unity when plotted against one another. The concordance coefficients for the shape descriptors are presented in Table 2 as a percentage in order to represent the similitude of the strain-step fields.

Finally, it is interesting to plot the residuals of the differences between feature vectors in order to observe if any of the shape descriptors show larger differences, which could give information about the evolution of the differences along the studied steps. 

**Figure 10:**
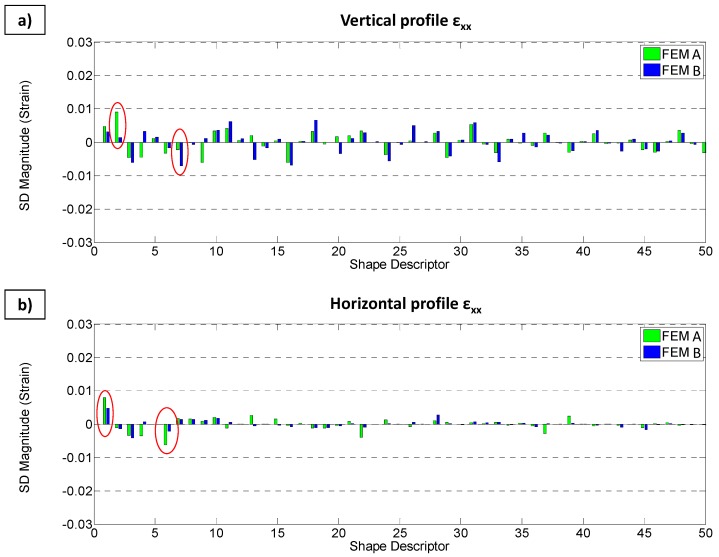

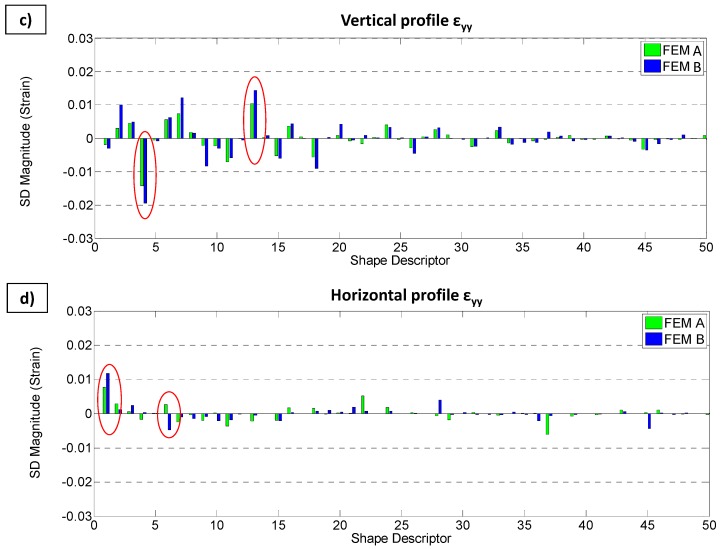
Residuals of the difference between experimental and numerical results (FEM A in green and FEM B in blue) for strain-step maps representing ε_xx_ along vertical (**a**) and horizontal (**b**) profile; and ε_yy_ along vertical (**c**) and horizontal (**d**) profile.

Figure 10 presents bar graphics of the residuals corresponding to the four strain-step maps (i.e., the vertical and horizontal profiles for ε_xx_ and ε_yy_ strains). The residuals are calculated by directly subtracting experimental and FEM feature vectors. The focus is placed on the two shape descriptors that offer maximum differences (red circles in Figure 10).

**Figure 11:**
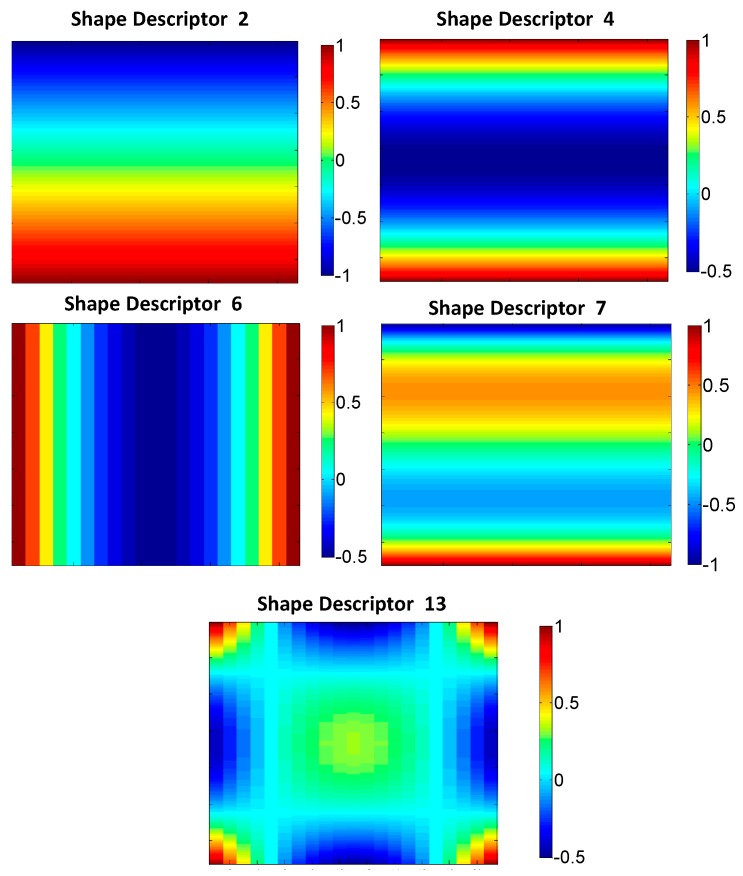
Illustration of the normalized kernels of the shape descriptors with bigger differences between experimental and numerical results.

It is clearly observed that differences in the shape descriptors values are very small. Figure 11 shows the kernels of the Tchebichef shape descriptors with higher differences. These kernels illustrate the distribution of the normalized residuals along the strain-step map in a two-dimensional representation. Thus, they also provide information about the evolution of the differences along the indentation. 

Bigger differences in ε_xx_ and ε_yy_ strain-step maps for horizontal profiles are observed in SD#1 and #6. In the case of ε_xx_ strain-step maps for the vertical profile, larger differences are observed in SD#2 and #7. Finally, in the case of ε_yy_ strain-step maps for vertical profile, larger differences are observed for SD#4 and #13. The kernels of those shape descriptors are presented in Figure 11. However, the kernel of SD#1 has a homogenous value indicating a full field offset in the strain value along the 21 studied steps, and it has not been considered in Figure 11.

## 4. Discussion

It is important to emphasize that the reduce area of interest and the high deformation level achieved during deformation made the calculation of the displacements maps using DIC complex. Additionally, as observed in Figure 7 and Figure 8, the indenter and the support of the specimen created some shadows and blind areas that made the visualization of some areas at the instant of maximum indentation difficult. To maximize the visualization of the whole area of interest during the full indentation and release, a specific optic set-up was required, as observed in Figure 2. With this optical arrangement, an uncertainty in the measurement of 31.7 µε was achieved when considering the similar set up employed by Tan et al. [2].

**Figure 12:**
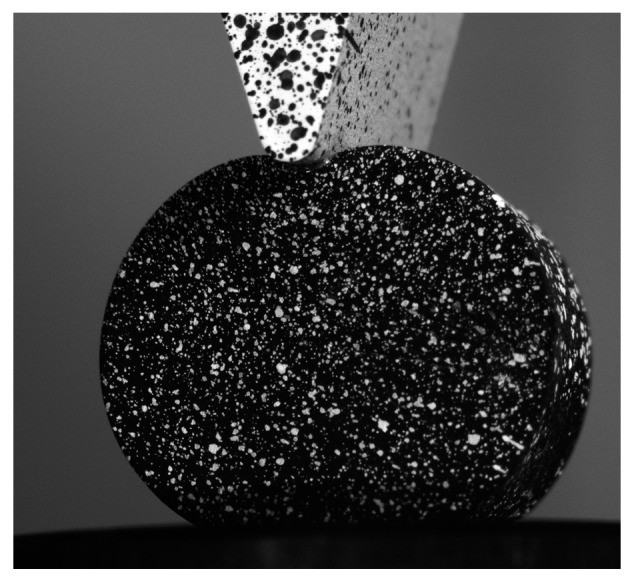
Image captured at maximum indentation instant.

Displacement maps obtained by this experimental procedure is presented in Figure 7. As observed, some negligible non-correlated areas are present (dark spots). It particular, some regions close to the indenter and at the lower area of the specimen were not able to be processed due to the high distortion, shadows, and the obstruction of the indenter in the cameras view, as shown in Figure 12. 

Those non-data areas are also present in experimental strain maps shown in the first row in Figure 8, where strains at maximum indentation are presented. Moreover, no large differences are observed by comparing those experimental and numerical results from Finite Element model A (and from Finite Element model B). However, it is observed that the FEM A model shows slightly lower maximum values in ε_xx_ (0.32 ε) and lower minimum values in ε_yy_ (0.30 ε), while the FEM B model presents slightly higher values in ε_xx_ (0.44 ε) and ε_yy_ (0.34 ε ) compare to experimental ε_xx_ and ε_yy_ values (0.39 and −0.25 strain, respectively).

Moreover, the focus is placed not only in point differences but in performing a full field comparison along the complete experiment. For this purpose, the strain-step images presented in Figure 9 show how the vertical and horizontal profiles of ε_xx_ and ε_yy_ varied along the studied step. Moreover, the creation of these strain-step maps allowed image decomposition to be performed through Tchebichef formulation, which is not applicable in non-rectangular data fields [19]. Moreover, it is observed that these strain-step maps agree between them. To quantify these differences, strain-step maps were decomposed into 50 feature vectors composed (Table 1). FEM model A obtained lower differences that model 2 in all the strain step maps, except for ε_xx_ in the horizontal profile strain map. Larger differences are observed for the ε_yy_ vertical profile strain map for the FEM B model, which represents a difference of 14.2% respect the maximum ε_yy_ experimental value compare to the 10% of difference obtained employing the FEM A model.

The correlation coefficients obtained in Table 2 show a value close to 100%, but again the results from FEM model A show higher concordance with experimental results. 

An interesting analysis can be also performed observing the differences in the kernels of the SD as illustrated in Figure 10 and Figure 11. 

Looking at the differences in ε_xx_ (Figure 10a), there are more important differences in SD#2 and SD#7 between experimental results and those obtained by the FEM A and FEM B models, respectively. From the representation of kernel of SD#2 in Figure 11, it is observed that the value of experimental strain in the upper area of the strain-step map is slightly bigger than the value of the FEM A model along all the steps of the indentation and, on the contrary, the value of experimental strain in the lower area of the strain-step map is slightly lower than the value of that FEM model. On the other hand, from the representation of kernel of SD#7, it is observed that differences between experimental and FEM B models are also present along the horizontal axis, which means that they are present along all indentation steps. It is also observed that in the upper area close to the edge of the strain-step map, the experimental value is bigger that the simulated value, while in the lower area of the image, the opposite occurs. This indicates that experimental deformation is slightly more focused in the contact area compare to the numerical results. Moreover, the FEM B simulation values are bigger than experimental results just above the half of the strain-step map and are lower just under it.

Differences in ε_xx_ (Figure 10b) suggest that the larger differences between FEM A and experimental results are associated on SD#1 and SD#6. As previously mentioned, SD#1 refers to a full field offset. In this case, the general experimental value of ε_xx_ horizontal profile is lower than the value of FEM A. Additionally, from the kernel of SD#6 in Figure 11, it is observed that an evolution of the difference exists, reaching it maximum values at the central area of the map, which implies the maximum indentation instant. That difference indicates that the values of experimental ε_xx_ are lower than FEM A results mainly at that instant.

With respect to the differences in ε_yy_ (Figure 10c), the differences between shape descriptors are slightly bigger than in the rest of the cases, as illustrated in Figure 10. These differences are more focused in SD#4 and #13 and between the experimental and FEM B results. Attending to the kernels of SD#4, a discordance along the whole indentation is observed where the vertical edges of the vertical profiles are bigger in the FEM B model rather than in the experimental results. Additionally, the values of experimental data in the central area are bigger than those of FEM model B.

Finally, bigger differences between both FEM models and experimental data are focused in SD#1, which means that both FEM models predicted a slightly bigger value in ε_yy_ along the horizontal profile than that which was measured experimentally (Figure 10d). However, this difference is larger for FEM B. SD#13 also presents some discrepancy to the central area of the strain-step map. This discrepancy is different for FEM A and FEM B models. In the case of FEM A, ε_yy_ was slightly larger than the experimental results, and the opposite happened for FEM B.

## 5. Conclusions

This paper presents a procedure to validate finite element models of indentations in soft materials. The validation was demonstrated by performing an experiment where a rubber cylinder (25 mm diameter and 15 mm high) was subjected to a lateral quasistatic indentation and subsequently released by a rounded edged rigid indenter. Three-Dimensional Digital Image Correlation was employed to calculate three-dimensional displacement fields from which strain maps were calculated. Two finite element models were developed to simulate the experimentation according to two different material models, i.e., Neo Hooken (FEM A) and Van der Walls (FEM B). The validation of those models was performed by comparing strain fields along the complete indentation process by employing image decomposition.

The adopted validation procedure is based on a quantitative comparison of the strain fields in X and Y directions. ε_xx_ and ε_yy_ strain maps for each of the studied steps (21 ε_xx_ strain map and 21 ε_yy_ strain map for each model and experimentation procedure) were combined into two strain-step maps for each procedure. Those strain-step maps where quantitatively compared by employing an Image Decomposition method based on Tchebichef shape descriptors. This methodology quantified differences between experimental results and models that were lower than 10% in case of FEM A. Correlation values between experimental and predicted values were always above 93%, and in the case of FEM A, the correlation factor achieved 97.1%. In addition, differences between strain fields were evaluated along the evolution of the indentation through the study of the kernels of the Shape Descriptors, which offered more important differences.

The validation procedure concluded that the FEM A model (Neo Hookean) better predicts the experimental data than the FEM B model (Van der Walls), which agrees with previous estimations [26].

The quantitative validation approach to comparing strain fields presented herein could provide an intervention methodology to easily validate dynamic (even at high speed) indentation models, enormously reducing the data sets. Additionally it is suitable in cases where the specimen studied is not rectangular, avoiding certain restrictions of some Image Decomposition procedures.

## Figures and Tables

**Table 1:** Euclidean distances between feature vectors representing strain-step maps.

-	FEM A (Strain)	FEM B (Strain)
ε_xx_ Vertical profile strain map	0.0211	0.0236
ε_xx_ Horizontal profile strain map	0.0137	0.0096
ε_yy_ Vertical profile strain map	0.0252	0.0356
ε_yy_ Horizontal profile strain map	0.0138	0.0152

**Table 2:** Correlation coefficient between feature vectors representing strain-step maps.

-	FEM A (Correlation Coef. in %)	FEM B (Correlation Coef. in %)
ε_xx_ Vertical profile strain map	98.1%	97.7%
ε_xx_ Horizontal profile strain map	98.3%	98.4%
ε_yy_ Vertical profile strain map	96.7%	93.3%
ε_yy_ Horizontal profile strain map	95.3%	94.8%
